# Asociación entre los estilos de crianza y el rol de los adolescentes peruanos en el acoso escolar, 2019*

**DOI:** 10.15649/cuidarte.2679

**Published:** 2023-05-28

**Authors:** Roberto Zegarra-Chapoñan, Jhon Alex Zeladita-Huaman, Juana Matilde Cuba-Sancho, Henry Castillo-Parra, Gladys Ivonne Moran-Paredes, Lucila Cárdenas-Niño

**Affiliations:** 1 . Facultad de Ciencias de la Salud, Universidad María Auxiliadora, Lima, Perú. Email: rob.zegarra@gmail.com Universidad María Auxiliadora Facultad de Ciencias de la Salud Universidad María Auxiliadora Lima Peru rob.zegarra@gmail.com; 2 . Facultad de Medicina, Universidad Nacional Mayor de San Marcos, Lima, Perú. Email: jhonzeladita@hotmail.com Universidad Nacional Mayor de San Marcos Facultad de Medicina Universidad Nacional Mayor de San Marcos Lima Peru jhonzeladita@hotmail.com; 3 . Facultad de Medicina, Universidad Nacional Mayor de San Marcos, Lima, Perú. Email: jcubas@unmsm.edu.pe Universidad Nacional Mayor de San Marcos Facultad de Medicina Universidad Nacional Mayor de San Marcos Lima Peru jcubas@unmsm.edu.pe; 4 . Facultad de Ciencias de la Salud, Universidad María Auxiliadora, Lima, Perú. Email: gerencia@neuromind.net Universidad María Auxiliadora Facultad de Ciencias de la Salud Universidad María Auxiliadora Lima Peru gerencia@neuromind.net; 5 . Facultad de Ciencias de la Salud, Universidad María Auxiliadora, Lima, Perú. Email: gladys.moran@uma.edu.pe Universidad María Auxiliadora Facultad de Ciencias de la Salud Universidad María Auxiliadora Lima Peru gladys.moran@uma.edu.pe; 6 . Departamento de Psicología, Universidad de San Buenaventura, Medellín, Colombia. Email: lucilacardenas1@gmail.com Universidad de San Buenaventura Departamento de Psicología Universidad de San Buenaventura Medellín Colombia lucilacardenas1@gmail.com

**Keywords:** Adolescente, Acoso Escolar, Crianza del Niño, Estudiantes, Adolescent, Bullying, Child Rearing, Students, Adolescente, Bullying, Educagáo Infantil, Estudantes

## Abstract

**Introducción:**

Los estilos de crianza determinan la conducta infantil; sin embargo, existe escasa información sobre su repercusión en el acoso escolar.

**Objetivo:**

Determinar la fuerza de asociación entre las diferentes tipologías de los estilos de crianza y el rol asumido en el acoso escolar, así como identificar perfiles de los adolescentes según su involucramiento en el acoso escolar.

**Materiales y métodos:**

Estudio transversal tipo analítico, realizado en Lima-Perú, en el año 2019. Se aplicó la escala de estilos de crianza de Steinberg y la escala EBIP-Q a 563 escolares de escuelas públicas. Los puntajes fueron obtenidos mediante un análisis factorial confirmatorio. Se empleó el coeficiente de correlación de Spearman, Chi Cuadrado de Pearson y análisis de regresión logística multinomial.

**Resultados:**

Existe asociación entre los estilos de crianza y el rol del adolescente en el acoso escolar. El análisis multivariado reportó que ser hombres se asocia con el rol de agresor (OR = 2,73); mientras que acceder a una red social (OR = 2,23), ser hombre (OR = 1,75), los estilos de crianza (negligente [OR = 2,72] y mixto [OR = 0,38] en comparación con autoritario) fueron predictores de asumir el rol de agresor victimizado en el acoso escolar.

**Discusión:**

La asociación encontrada en el estudio confirma hallazgos previos; sin embargo, se abre debate sobre la influencia del estilo de crianza negligente en la dinámica del acoso escolar.

**Conclusión:**

Tanto el estilo de crianza negligente y autoritario (menor proporción) tienen un efecto positivo y significativo en el rol de agresor victimizado.

## Abstract (en)

**Introduction:**

Parenting styles determine child behavior; however, there is little information on their impact on bullying.

**Objective:**

To determine the association between different parenting styles and the role assumed in bullying, as well as to identify adolescents' profiles according to their involvement in bullying.

**Materials and Methods:**

A cross-sectional analytical study was conducted in Lima, Peru, in 2019. The Steinberg's Parenting Styles Scale and the European Bullying Intervention Project Questionnaire (EBIP-Q) were administered to 563 students from public schools. Scores were obtained by confirmatory factor analysis. Spearman's correlation coefficient, Pearson's Chi-square test, and multinomial logistic regression analysis were used.

**Results:**

There is an association between parenting styles and adolescents' role in bullying. The multivariate analysis reported that being male was associated with the role of the bully (OR: 2.73; 95% CI 1.59-4.68), while access to social media (OR: 2.23; CI 95% 1.11-4.49), being male (OR: 1.75; CI=1.06-2.88), and parenting styles (neglectful [OR: 2.72; 95% CI 1.32-5.59] and mixed [OR: 0.38; 95% CI 0.16-0.91] compared to authoritarian parenting style) were predictors of the role of the victimized bully.

**Discussion:**

The association found in the study confirms previous findings; however, it opens the debate on the influence of neglectful parenting style on bullying dynamics.

**Conclusions:**

Both neglectful and authoritarian parenting styles (lower proportion) have a positive and significant effect on the role of victimized bully.

## Resumo

**Introdujo:**

Os estilos parentais determinam o comportamento das crianzas; no entanto, há pouca informado sobre o seu impacto no bullying.

**Objetivo:**

Determinar a forga da associagáo entre os diferentes estilos parentais e o papel assumido no bullying; bem como identificar perfis de adolescentes de acordo com seu envolvimento no bullying.

**Materiais e métodos:**

Estudo analítico transversal realizado em Lima-Peru em 2019. A escala de estilos parentais de Steinberg e a escala EBIP-Q foram aplicadas a 563 alunos de escolas públicas. Os escores foram obtidos por meio de uma análise fatorial confirmatória. Foram utilizados o coeficiente de correlagáo de Spearman, o Qui-Quadrado de Pearson e a análise de regressáo logística multinomial.

**Resultados:**

Existe associagáo entre os estilos parentais e o papel do adolescente no bullying. A análise multivariada relatou que ser do sexo masculino está associado ao papel de agressor (OR: 2,73; IC 95% 1,59-4,68); ao acessar uma rede social (OR: 2,23; IC 95% 1,11-4,49), ser do sexo masculino (OR: 1,75; IC=1,06-2,88), estilos parentais (negligente [OR: 2,72; IC 95% 1,32-5,59] e misto [OR: 0,38; IC 95% 0,16-0,91] comparado ao autoritário) foram preditores de assumir o papel de agressor vitimizado no bullying.

**Discussao:**

A associagáo encontrada no estudo confirma achados anteriores; no entanto, abre-se um debate sobre a influencia do estilo parental negligente na dinámica do bullying.

**Conclusao:**

Tanto o estilo parental negligente quanto o autoritário (menor proporgáo) tem efeito positivo e significativo no papel do agressor vitimizado.

## Introducción

El establecimiento de las relaciones sociales se gesta desde el hogar, por ello las ciencias del comportamiento y las ciencias sociales señalan como un hecho fundamental la importancia de la calidad de la familia en el desarrollo emocional de los niños^1^; es decir la forma como los padres dirigen y gestionan el comportamiento del hijo, la expresión del afecto, su implicación en el manejo de las emociones de estos, juega un papel fundamental en la creación del vínculo afectivo de identificación y el desarrollo emocional y cognitivo de los seres humanos. Las figuras parentales primarias son claves para establecer patrones sanos de confianza y comportamiento prosocial de los niños en las relaciones con los otros y en gran medida se teoriza que esto está mediando por los patrones de apego temprano entre padres e hijos y determina las estrategias de ajuste emocional del niño con su entorno social y el grado de afiliación con sus redes sociales^2^.

El estilo de apego se refiere al nivel de seguridad que tiene un niño en sus etapas iniciales del desarrollo con su madre o su padre, los apegos de tipo seguros reflejan una crianza cálida y receptiva y permiten que el niño se mueva en el mundo con pro-socialidad y confianza^3^. Los apegos de tipo ansiosos reflejan inconsistencia en las respuestas de los padres cuando el niño tiene necesidades emocionales o está estresado y “dejan al niño ansioso de su propio valor y competencia” ^4^. Los apegos de tipo evitativos reflejan la falta de calidez emocional y de apoyo afectivo de parte de los padres y generan en el niño sentimientos de que debe cuidar a sí mismo y la forma en que se relacionará con otros niños^5^.

La escuela es el espacio donde estas relaciones sociales se consolidan en su relación con sus pares; y donde otro fenómeno: el acoso escolar toma especial interés para la sociedad. El acoso y la victimización se clasifican en términos generales como tradicionales y ciberacoso. El acoso tradicional se refiere a “cualquier comportamiento agresivo no deseado por parte de otro niño o grupo de jóvenes que implica un desequilibrio de poder y se presenta de forma repetitiva”; mientras que en el ciberacoso esta conducta es mediada por la tecnología^6^. Diversos estudios han confirmado que el acoso escolar, en cualquiera de sus formas impactan negativamente en el rendimiento escolar^7^y el bienestar del niño y adolescente en las diferentes esferas de funcionamiento vital a nivel social, fisiológico y psicológico^8^.

La Organización Mundial de la Salud reportó que la tercera parte de la población escolar experimentó acoso tradicional en el periodo de un mes^9^. De manera similar, en Colombia un estudio realizado en estudiantes destaca que el 38,5% fue víctima de acoso escolar^10^. Asimismo, en Perú, un estudio que analizó información de un sistema de auto-reporte sobre casos de violencia escolar, señaló que su incidencia en todas sus formas se incrementó entre los años 2014 a 2018^11^.

Estudios realizados en países latinoamericanos confirman que las características individuales como la edad y el sexo están asociados a las actitudes hacia la violencia^12^ y el acoso escolar^10^; sin embargo, las sociedades actuales consideran cada vez más a los padres como responsables moral y legalmente por el comportamiento inadecuado de sus hijos y cuestiona a estos en su aptitud parental si sus hijos son agresores o victimizados en una situación de acoso escolar.

Aunque no son muchos los estudios que han analizado la relación entre los estilos de crianza y el papel de agresor o la víctima, hay un acuerdo en la comunidad científica de que los padres autoritativos operan como factores reductores del acoso tradicional tanto a nivel de agresor o de victimización^13^^-^^15^; de forma contraria, se observa que el estilo de crianza autoritario se asocia directamente con el involucramiento en el acoso tradicional^13^^,^^14^^,^^16^^,^^17^. En esta misma línea, un estudio longitudinal que realizó seguimiento a un grupo de padres que inicialmente tenían un estilo de crianza autoritario y luego adoptaron el estilo autoritativo después de una intervención, sus hijos mostraron un incremento en el autoconcepto y disminución de la conducta agresiva^18^.

No obstante, una revisión sistemática de la literatura, sobre la relación entre ciertos estilos parentales y la presencia de acoso tradicional reportó que solo en cuatro de 10 de dichos estudios primarios encontraron una relación estadísticamente significativa entre el estilo parental autoritario y la conducta de acosador en sus hijos y que solo cuatro de ocho investigaciones identificaron una asociación significativa entre estilos parentales autoritativos y victimización^19^. Estos resultados sugieren que los hallazgos nulos son al menos tan probables como los hallazgos de que el estilo de crianza afecta la participación en el acoso tradicional.

Por otra parte, los resultados sobre la relación entre la crianza indulgente y la victimización por acoso tradicional son igualmente mixtos; algunos estudios concluyeron que la crianza indulgente se asocia con la probabilidad más alta^15^^,^^17^^,^^20^ mientras que otra investigación discrepa al señalar que no existe asociación entre la paternidad indulgente y la victimización en el acoso tradicional^13^. En otro estudio^21^, se investigó la relación entre el tipo de apego desarrollado por los niños hasta su adultez y los estilos de crianza de parte de los padres; se evaluaron 74 adultos con Adult Attachment Questionnaire y el cuestionario de estilos de crianza cuyo resultado muestra una correlación significativa entre el apego adulto seguro y el estilo de crianza autoritario.

Sobre los estilos de crianza, aquellos donde los padres son autoritativos están caracterizados por su orientación racional, exigente con el cumplimiento de reglas como estándar de comportamiento para establecer relaciones asertivas pues los escuchan, son afectuosos (dar-tomar) y tratan de manera cálida pero siempre al pendiente de su actuar; al contrario de ellos, los padres autoritarios no expresan afecto sino se imponen y reafirman su control usando la fuerza física, suelen exigir, demandar y dirigirlos invadiendo su espacio. Por su parte los padres permisivos, también llamados indulgentes o no directivos, no establecen normas e interfieren poco en las decisiones de sus hijos, se muestran afectuosos y emplean la razón o persuasión evitando confrontarlos; mientras que los padres negligentes no suelen establecer límites o no les interesa asumir su rol frente a ellos, tampoco expresan afecto^22^.

Es clara, la fuerte relación que hay entre los estilos parentales, los tipos de apego relacionado y las formas de socialización que los niños desarrollaran posteriormente en la escuela y otros escenarios sociales; los padres como responsables de sus hijos, son actores centrales en los esfuerzos por prevenir y reducir al acoso^23^; sin embargo, desde una perspectiva comparativa, son pocos los estudios que se han enfocado en describir y explicar las dinámicas de la relación entre estilos de crianza y el papel asumido en las experiencias de acoso escolar^13^^,^^17^. Además, tales estudios han arrojado hallazgos no concluyentes o resultados contradictorios, que justifican el presente estudio. En ese sentido, la presente investigación busca determinar la fuerza de asociación entre las diferentes tipologías de los estilos de crianza y el rol asumido en el acoso escolar, así como identificar perfiles de los adolescentes según su involucramiento en el acoso escolar.

## Materiales y Métodos

### Tipo de estudio

Estudio transversal de tipo analítico realizado entre agosto a diciembre del 2019 en escolares de educación básica regular. Fue guiado por la herramienta STROBE.

### Participantes

La población estuvo conformada por 1 200 escolares de 10 instituciones educativas públicas (estatales) ubicadas en 9 distritos de Lima Metropolitana, Perú. Considerando que el grupo de interés a estudiar eran los adolescentes; se optó como criterios de inclusión: estar matriculados entre el sexto grado de primaria y el tercer año de secundaria y tener entre los 11 a 16 años. Se excluyeron a quienes presentaban alguna discapacidad mental que no permitiera completar el formulario.

La muestra mínima (149 escolares) fue calculada considerando un 95% de nivel de significancia y 5% de margen de error. Sin embargo, en el estudio se incluyeron 564 escolares; quienes fueron seleccionados mediante muestreo no probabilístico por conveniencia. Es necesario mencionar que el Ministerio de Educación del Perú emprendió una política nacional orientada a incrementar el tiempo efectivo en la tarea escolar; por ello, las comunidades docentes se han tornado más reticentes a facilitar el ingreso de investigadores a los recintos escolares y la razón que explica el tipo de selección de muestra para este estudio. Del mismo modo, el interés primordial de esta investigación fue el análisis de la asociación entre las variables de interés más en una línea relacionada con el avance teórico comprensivo de este fenómeno; lo cual nos aleja en cierta medida de los estudios que pretenden establecer incidencias y/o prevalencias en una población. En tanto, este tipo de muestreo contempló el salvaguardar la existencia de estratos con similar proporción en cuanto a hombres - mujeres. Igualmente, se procuró que todas las escuelas se parezcan entre sí en cuanto al estrato que atienden, pero al mismo tiempo, que presenten diferencias marcadas por el distrito al que pertenecen.

### Instrumento y Variables

Se empleó un cuestionario autoadministrado conformado por tres secciones. En la primera, se recolectaron datos sociodemográficos del adolescente (edad en años cumplidos y sexo), tipo de núcleo de convivencia familiar, tenencia de redes sociales, año de estudios y si había recibido alguna orientación o capacitación sobre acoso escolar.

En la segunda sección, para determinar los estilos de crianza se empleó la Escala desarrollada por Lawrence Steinberg que fue validada en población peruana (Alfa de Cronbach de 0,74)^22^. Adicionalmente, con los datos recopilados, se realizó un análisis psicométrico que fue publicado previamente en otro manuscrito, en donde se confirmó que la escala presenta buenas propiedades psicométricas en una muestra de adolescentes peruanos (índice Omega McDonald = 0,944)^24^. La escala empleada estuvo conformada por 21 reactivos agrupados en tres dimensiones: compromiso (9 ítems), autonomía psicológica (6 ítems) y control conductual (6 ítems). Las preguntas de las dos primeras dimensiones constan de 4 alternativas de respuesta, que va desde 1 (muy en desacuerdo) hasta 4 (muy de acuerdo). La tercera dimensión consta ítems que tienen 3 opciones, que van desde 1 (no tratan) hasta tres (tratan bastante).

En la última sección, para identificar el rol que asumen los adolescentes en el acoso se empleó el European Bullying Intervention Project Questionnaire (EBIPQ por sus siglas en inglés). De manera similar, con los datos recopilados, mediante análisis factorial exploratorio, que fue publicado previamente en otro manuscrito^25^, se confirma que la estructura factorial de esta escala en escolares peruanos es similar al cuestionario original y reporta elevada confiabilidad y validez (alfa de Cronbach de 0,856). Los ítems de esta escala tipo Likert corresponden a aspectos relacionados con la agresión y victimización, cuyas alternativas de respuesta mide la frecuencia desde 0 = “Nunca” hasta 4 = “Siempre”.

### Procedimientos

Previa la recopilación de datos, se realizó una prueba piloto en 25 estudiantes de una institución educativa de similares características con la finalidad de evaluar las condiciones de aplicación y comprensión de los ítems por los adolescentes.

Las fechas para la recolección de datos se establecieron en coordinación con el director de cada institución educativa.

Al inicio de la recolección de datos, se reiteró la importancia de su colaboración. Además, a los escolares se les indicó que no hay respuestas buenas, ni malas, ni correctas o incorrectas. Luego, en el aula de clases se aplicó el cuestionario a cada estudiante. El tiempo que empleaban los participantes para completar el cuestionario fue de 20 minutos en promedio.

### Aspectos éticos

El estudio fue aprobado por el Comité de Ética de la Universidad María Auxiliadora (Constancia N° 09 2019). Previa la recopilación de datos se realizó el proceso de consentimiento informado a los padres o apoderados y luego el proceso de asentimiento informado a los adolescentes, tal como establece la regulación peruana.

### Procesamiento estadístico

Luego de realizar su validación, la base de datos del presente estudio debidamente organizado se presenta en un archivo Excel versión para Windows 2010 y está disponible en el Data-set^26^. Se realizó análisis descriptivos de frecuencias y porcentajes (variables categóricas) medias, desviaciones estándar (variables continuas). Para identificar los puntajes de las dimensiones en las variables investigadas se utilizaron los puntajes factoriales obtenidos de un análisis factorial confirmatorio; con estos puntajes se procedió a realizar correlaciones entre todas las dimensiones mediante el Coeficiente de Correlación de Spearman. Por otro lado, se procedió a categorizar los estilos de crianza (autoritativo, negligente, autoritario, permisivo indulgente y mixto) y las categorías de roles asumidos en el bullying (agresor, victima-agresor, victima, no implicado). Para el análisis bivariado se empleó la prueba de Chi Cuadrado de Pearson. Adicionalmente, se realizó un análisis de correspondencia entre ambas variables. Para determinar la fuerza de asociación se procedió a realizar un análisis de regresión logística multinomial con el rol asumido en el bullying como variable de respuesta; mientras que, las variables sociodemográficas y los estilos de crianza fueron tomados como variables predictoras. Finalmente, se realizó un análisis de cluster para caracterizar los adolescentes según el rol que asumieron en el acoso escolar. En nivel de significancia fue de 0,05. Los análisis del presente estudio se llevaron a cabo en R v.4.0.0, SPSS y XLSTAT.

## Resultados

Participaron 563 adolescentes cuya edad media fue 12,96 años (DE = 1,31), de los cuales el 54,0% (304) fueron mujeres y el 66,85% (373) vivía con ambos padres. Las otras características sociodemográficas y familiares se describen en la Tabla 1.

**Tabla 1:** Características sociodemográficas y familiares de los adolescentes (n=563). Lima, 2019

Características	n	%
Sexo		
Masculino	259	46,01
Femenino	304	53,99
Convivencia familiar*		
Con su padre	26	4,65
Con su madre	141	25,27
Con su padre y madre	373	66,85
Con otros familiares	18	3,23
Tiene una cuenta en alguna red social**		
No	106	19,20
Sí	446	80,80
Año de estudio		
Sexto de primaria	159	28,24
Primero de secundaria	165	29,31
Segundo de secundaria	137	24,33
Tercero de secundaria	102	18,12
Recibió orientación sobre acoso escolarf		
No	166	30,07
Sí	386	69,93

* Solo respondieron 558 alumnos; t Solo respondieron 552 alumnos

El estilo de crianza que predomina entre los adolescentes encuestados fue el de padres permisivos indulgentes con 28,42% (160), el siguiente estilo más frecuente en la muestra fue el de padres autoritarios con 22,56% (127). Por otro lado, el porcentaje más bajo se observa para quienes tiene padres autoritativos con 11,55% (65), mientras que los padres mixtos y negligentes representan el 18,65% (105) y 18,82% (106), respectivamente.

Con respecto a los roles asumidos en el acoso escolar, se observa que los no implicados representan el 40,32% (227) de la muestra total. En cuanto a los que se involucran, los adolescentes víctimas representan el 25,04% (141); los agresores victimizados, el 18,65% (105) y finalmente, el grupo más pequeño fue el de agresores que representa el 15,99% (90).

### Correlación entre las dimensiones de estilos de crianza y el acoso escolar

A medida que el padre tiene un mayor “Compromiso” se presentan menores conductas de agresor (Rho = -0,12) y menores conductas de victimización (Rho = -0,16). Por otro lado, en medida que el padre ejerce más “Control” el hijo tiene menos conductas agresivas (Rho = -0,14). Finalmente, los padres que entregan más “Autonomía”, tienen hijos que muestran más conductas de victimizados (Rho = 0,19) (Tabla 2).

**Tabla 2:** Matriz de correlaciones de los puntajes factoriales de las dimensiones de los estilos de crianza y roles asumidos en el acoso escolar por los adolescentes (n=563). Lima, 2019

Dimensión	1	2	3	4	5
1. Estilo de crianza: dimensión compromiso	-				
2. Estilo de crianza: dimensión control	-0,03	-			
3. Estilo de crianza: dimensión autonomía	-0,05	0,01	-		
4. Acoso escolar: dimensión agresor	-0,12*	-0,14t	0,05	-	
5. Acoso escolar: dimensión victimización	-0,16t	-0,08	0,19t	0,02	-

* valorp < 0,01; t valor p < 0,001

### Relación entre los estilos de crianza y el rol en el acoso escolar

**Figura 1:**
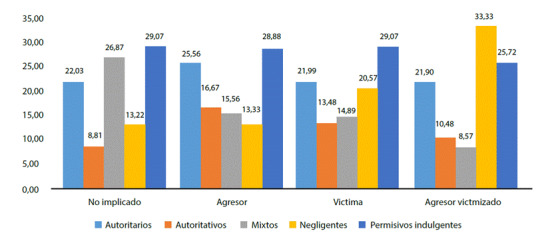
Porcentaje de estilos de crianza en cada uno de los roles asumidos en el acoso escolar por los adolescentes (n=563). Lima, 2019

El análisis bivariado mediante la prueba Chi Cuadrado de Pearson (Figura 1) determinó que los porcentajes de los adolescentes según el estilo de crianza se encuentran asociadas con los roles que asumen en el acoso escolar. (X2 = 37,51, gl= 12, p < 0,001). Específicamente, se observa que el porcentaje de adolescentes criados por padres permisivo indulgente predomina entre quienes no se involucran en el acoso escolar (29,07%), entre los agresores (28,88%) y entre los que fueron víctimas (29,07%); sin embargo, destaca el estilo de crianza negligente entre los escolares que asumieron el rol de agresor victimizado (33,33%).

**Figura 2:**
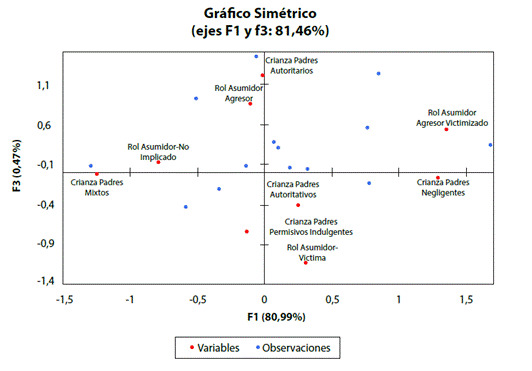
Análisis de correspondencias entre los estilos de crianza y el rol asumido en el acoso escolar

El análisis de correspondencias establece la relación entre variables categóricas y las dos tipologías de estilos de crianza y el rol asumido por el adolescente en el acoso escolar, logrando reducir a 3 componentes principales con una varianza acumulada del 99%. Las características de los escolares analizados bajo esta metodología se encuentran resumidos en la Figura 2.

### Asociación entre las diferentes tipologías de los estilos de crianza con el rol que asumen en el acoso escolar

En la Tabla 3 se pueden observar los resultados de la regresión logística multinomial para identificar las características de los perfiles de roles asumidos en el acoso escolar. En la primera parte del modelo en cuanto a la comparación de agresores versus no implicados, solo se observa una asociación significativa en cuanto al sexo, específicamente los hombres, quienes presentan una probabilidad de 2,73 veces mayor de ser categorizados como agresores.

No se observaron asociaciones significativas en la probabilidad de ser clasificado como víctima versus no implicado. Por otro lado, en la parte del modelo que compara las probabilidades de ser categorizado como no implicado versus agresor victimizado se observa que los hombres tienen 1,75 (p < 0,05) veces mayor probabilidad que las mujeres para ser categorizado como agresor victimizado. Del mismo modo, los adolescentes que tienen alguna red social tienen 2,23 (p<0,05) veces mayor probabilidad de ser categorizado como agresor victimizado que aquellos que no tienen una red social.

En cuanto a los estilos de crianza, los hijos de padres con un estilo de crianza mixto tienen probabilidades 62% (p < 0,05) más pequeñas de ser categorizados como agresores victimizados que los hijos de padres con un estilo de crianza autoritario. Finalmente, los hijos con padres negligentes tienen 2,72 (p<0,01) veces mayor probabilidad de ser categorizados como agresores victimizados que los hijos de padres autoritarios.

**Tabla 3:** Modelo de regresión logística multinomial sobre los roles asumidos en el acoso escolar, usando el rol no implicado como categoría de referencia, para los adolescentes (n=563). Lima, 2019

Características	No implicado vs Agresor		No implicado vs Victima		No implicado vs Agresor Victimizado	
	OR	[OR: IC 95%]	OR	[OR: IC 95%]	OR	[OR: IC 95%]
Sexo						
Femenino	1		1		1	
Masculino	2,73$	[1,59 - 4,68]	0,96	[0,63 - 1,49]	1,75t	[1,06 - 2,88]
Tenencia de una red social	1,23	[0,66 - 2,32]	1,25	[0,73 - 2,15]	2,23t	[1,11 - 4,49]
Recibió orientación sobre	0,69	[0,41 - 1,16]	1,28	[0,79 - 2,07]	1,46	[0,84 - 2,54]
acoso escolar (sí)						
Estilo de crianza						
Padres Autoritarios	1		1		1	
Autoritativo	1,39	[0,59 - 3,26]	1,52	[0,7 - 3,31]	1,10	[0,44 - 2,77]
Mixto	0,52	[0,24 - 1,13]	0,58	[0,3 - 1,14]	0,38t	[0,16 - 0,91]
Negligente	0,75	[0,32 - 1,76]	1,49	[0,74 - 2,98]	2,72*	[1,32 - 5,59]
Permisivo indulgente	0,78	[0,39 - 1,55]	1,06	[0,58 - 1,95]	0,98	[0,49 - 1,95]

*valor p < 0,01; tvalor p < 0,05; t valor p < 0,001; § el estilo de crianza de referencia fueron los padres autoritarios.

En el proceso de clusterización se construyen 4 clusters definitivos, el primero de ellos en donde se agrupan 148 adolescentes con un peso del 26,29% de la muestra. En este cluster se ubicaron escolares con características similares cómo: quienes no se implicaron en el acoso escolar, tenían un estilo de crianza de padres mixto o estilos de crianza de padres permisivos indulgentes, la mayoría de los individuos de este cluster fueron mujeres (valor p< 0,05). Las demás variables incluidas dentro del cluster no fueron estadísticamente significativos. El segundo cluster se conformó con 60 adolescentes con un peso de 10,66% de la muestra y se caracterizó por escolares que fueron víctimas de acoso escolar (100%), estilos de crianza de padres permisivos indulgentes (68,33%) y autoritativos (31,67%; valor p<0,05).

El tercer cluster se conformó por 202 adolescentes con un peso de 35,88% de la muestra, los cuales presentaron un rol en el acoso escolar mayoritariamente agresor (100%) con padres con estilos de crianza autoritarios y autoritativos y de sexo masculino. Todas las variables descritas fueron significativas (valor p<0,05). Finalmente, el cuarto cluster se conformó por 153 adolescentes con un peso de 27,18% de la muestra, compuesto mayoritariamente por quienes asumieron el rol de agresores victimizados, presentaban principalmente estilos de crianza de padres negligentes (valor p<0,05).

## Discusión

En este estudio con la finalidad de explorar la fuerza de asociación entre cada tipología de estilo de crianza con el acoso escolar se realizaron diferentes análisis estadísticos que confirmaron la asociación entre los estilos de crianza y el rol que asumen los adolescentes en el acoso escolar. Hallazgo que coincide con el consenso científico respecto a la influencia de los estilos de crianza en la conducta que asumen los adolescentes en la dinámica de la violencia escolar^17^,^27^ ^29^.

Cabe destacar que los estilos parentales afectan el sentido de autonomía y confianza emocional de los niños al tiempo que se han demostrado asociaciones significativas entre los estilos parentales de parte de los padres y los comportamiento sociales posteriores en los niños; aunque actualmente no existe una teoría definitiva sobre cómo los padres a través del tipo de apego generado con sus hijos y sus estilos de crianza, dan forma al desarrollo emocional de estos y como posteriormente afectara las habilidades de socialización posterior en estos niños^5^.

De manera específica, el estilo de crianza negligente se asocia con el rol de agresor victimizado. Este resultado sugiere que los hijos de padres negligentes, que no brindan calidez afectiva ni imponen disciplina firme, cuando son víctimas del acoso escolar suelen externalizar una conducta agresiva con sus pares. Esto podría ser deberse a que este estilo de crianza implica un bajo nivel de cubrimiento de las necesidades básicas emocionales de los hijos, un descuido significativo del rol de padre y se asocia con deterioro de las relaciones entre padres e hijos y problemas en el desarrollo infantil como la disminución de habilidades emocionales^30^ y con la presencia de problemas de externalizantes como agresividad^31^, la rebeldía y la propensión a presentar dificultades en las interacciones sociales^22^.

Asimismo, los hijos de padres de estilo de crianza mixto en comparación con quienes fueron criados en un estilo autoritarios tienen menor probabilidad de ser categorizados como agresores victimizados. Al respecto, un estudio longitudinal reportó que la adopción de un estilo de crianza autoritario se asocia con un incremento de la conducta agresiva y disminución del autoconcepto^18^.

Los padres autoritarios son rígidos y desproporcionados al imponer las normas, proyectan el poder sin miramientos y suelen ser fríos emocionalmente; lo que generan en el adolescente conductas agresivas y menos solidarias con sus pares^32^. Por último, respecto a las características sociodemográficas, en análisis multivariado reportó que los hombres tienen mayor probabilidad de asumir el rol de agresor victimizado. Hallazgo que coincide con un estudio que reporta que el género se asocia con el rol de víctima-agresor, pero no con el rol de víctima y agresor puro^33^.

El perfil del escolar agresor se caracteriza por agrupar adolescentes hombres que tuvieron un estilo de crianza autoritario o autoritativo. Respecto al género, el análisis de regresión confirmó que los hombres tienden a ser más agresivos, hallazgo que coincide con estudios previos^16^. De manera complementaria, el análisis de correspondencia, reportó que el rol de agresor se asocia al estilo de crianza autoritario: en concordancia, con investigaciones que relacionan el estilo de crianza autoritario con el comportamiento en los hijos de intimidación como acosadores^17^ y que tiene un efecto negativo en el acoso tanto tradicional o ciberacoso^13^. Además, la agresión en adolescentes se asocia de manera directa con el estilo de crianza autoritario y de manera inversa con tener padres autoritativos^14^; debido a que tienden a emplear prácticas agresivas, punitivas y métodos que promueven la aceptación de la violencia física y psicológica como una forma de enfrentar los problemas y resolver los conflictos interpersonales^13^.

En este estudio, el cluster conformado por adolescentes víctimas, mayoritariamente fueron hijos de padres permisivos indulgentes o de padres autoritativos. Asimismo, el análisis de correspondencia evidenció que el estilo de crianza de padres permisivos indulgentes se asocia con este rol. Hallazgo en línea con estudios previos^17^^,^^20^ y que coincide parcialmente con otro estudio, que reportó que la victimización se asocia con el estilo de crianza autoritativo, pero no con el estilo de crianza indulgente^13^.

Cabe señalar, que los autores del último estudio, en el que no encontraron asociación entre el estilo de crianza indulgente con la victimización, atribuyen los cambios importantes realizados a la escala y que los ítems reportaron una baja confiabilidad. Este hallazgo evidencia que tener padres permisivos, es decir que tienen un trato cálido, pero sin disciplina, pone al adolescente en riesgo de ser víctima de acoso escolar, sobre todo porque una de sus características es la sobreprotección^20^.

El análisis correlacional entre las dimensiones de las variables, confirma que el “Compromiso” en los padres reduce las conductas de agresión y victimización. De modo que los hijos de padres que puntúan alto en la capacidad de respuesta afectiva y emocional, tienen menos probabilidades de haber sido acosadores o victimas^15^^,^^34^. Adicionalmente, el “Control” reduce las conductas de agresión y la “Autonomía” aumenta la victimización. Sin embargo, la evidencia sobre esta asociación es mixta. Mientras que algunos estudios señalan como factor de riesgo de acoso a la baja exigencia^15^, otros reportan a los altos niveles de control y exigencia del comportamiento^35^. No obstante, los padres altamente receptivos brindan más protección contra el acoso que los muy exigentes^36^.

Desde el punto de vista metodológico, este es uno de los primeros estudios que combina diferentes métodos de análisis estadísticos complementarios que permitió obtener resultados más robustos. En primera instancia, producto del análisis factorial confirmatorio se estimaron puntajes factoriales para cada una de las dimensiones. La primera exploración de asociación entre los estilos de crianza y el rol en el acoso escolar se realizó mediante la prueba estadística de Chi Cuadrado de Pearson. Para identificar la tendencia de cada tipología de los estilos de crianza con el acoso escolar, primero se realizó un análisis de correspondencia, luego se complementó mediante regresión logística multinomial en el cual se conformaron tres modelos y que incluyó variables mediadoras como el sexo, tenencia de una red social, tipo de familia. Finalmente, se realizó un análisis de clasterización que permitió agrupar la muestra en cuatro grupos o clúster.

El estudio tuvo algunas limitaciones. El tipo de muestreo empleado no permite generalizar los resultados a otras poblaciones. El método de recolección de datos (auto-reporte) puede generar que los adolescentes marquen sus respuestas sin una lectura minuciosa; sin embargo, el equipo de recopiladores del estudio estuvo capacitado para identificar este tipo de conducta y pedirle al adolescente que lea antes de responder.

## Conclusiones

Aunque el estudio presentó algunas limitaciones desde el punto de vista metodológico, esta investigación integró diferentes métodos de análisis estadísticos complementarios que permitió la obtención de resultados robustos. El principal hallazgo del estudio es que existe asociación entre los estilos de crianza con el rol que asumen los adolescentes en el acoso escolar y que el estilo de padres “Negligentes” se asocia de forma significativa con el desarrollo de adolescentes clasificados como agresores victimizados.

Dentro de otros resultados obtenidos por la presente investigación se encontró adicionalmente que el “Compromiso” se asocia con la reducción de las conductas de agresión y victimización, el “Control” se asocia con la reducción de conductas de agresión y la “Autonomía” se asocia con el aumento de la victimización. En cuanto a los perfiles, los hombres tienden a ser más agresores y agresores victimizados que las mujeres. Asimismo, quienes usan redes sociales tienden a ser más agresores victimizados. Finalmente, el perfil de agresor victimizado se caracteriza por agrupar más hijos de padres autoritarios y en mayor medida padres negligentes.

Responsabilidades Éticas: Confidencialidad de los datos: Los autores declaran que han seguido los protocolos de su institución laboral sobre la publicación de datos de pacientes. Derecho a la Privacidad y Consentimiento Informado: Los autores declaran que realizaron el proceso de consentimiento informado a los padres o tutores legales. Asimismo, se realizó el asentimiento informado a los escolares.
